# Improving oral sentence production in children with cochlear implants: effects of equivalence-based instruction and matrix training

**DOI:** 10.1186/s41155-018-0095-y

**Published:** 2018-06-22

**Authors:** Anderson Jonas das Neves, Ana Claudia Moreira Almeida-Verdu, Grauben José Alves de Assis, Leandra Tabanez do Nascimento Silva, Adriane Lima Mortari Moret

**Affiliations:** 1 Universidade Federal de São Carlos, UFSCar, São Carlos, SP Brazil; 2 Universidade Estadual Paulista, UNESP, Bauru, SP Brazil; 3 Universidade Federal do Pará, Belém, PA Brazil; 4 Hospital de Reabilitação de Anomalias Craniofaciais, HRAC, Bauru, SP Brazil; 5 Universidade de São Paulo, USP, Bauru, SP Brazil; 6 Avenida Engenheiro Luiz Edmundo Carrijo Coube, 14-01 - Vargem Limpa, Bauru, SP 17033-360 Brazil

**Keywords:** Equivalence-based instruction, Sentences, Speech accuracy, Cochlear implant

## Abstract

**ᅟ:**

Children who use cochlear implants (CI) and who are readers usually produce more accurate speech in response to text than to pictures. Equivalence-based instruction (EBI) can be a route to establish functional interdependence between these verbal operants. The present study investigated whether children with CI who read would improve speech accuracy when tacting pictures of scenes after EBI that included dictated sentences, pictures of scenes, and printed sentences. This study evaluated whether teaching verbal relations to diagonal sentences from a matrix with subject-verb-object combinations promoted recombinative generalization to untrained sentences. Participants were three children with CI with a more accurate speech when reading print than when tacting pictures of scenes. They were taught to select pictures of scenes in response to dictated sentences (AB) by matching-to-sample (MTS) and to construct printed sentences in response to dictated sentences (AE) by constructed-response-matching-to-sample (CRMTS). Speech production in response to print (CD) and in response to pictures of scenes (BD) were probed for both trained and untrained sentences, using a multiple baseline design across participants. All participants learned the trained relations, showed emergence of derived relations, and improved speech accuracy when tacting pictures of scenes. They were able to recombine sentence components and tact novel pictures using untrained sentences from the matrix. These results indicate that speech accuracy and generative sentence production can be improved in children with CI from interventions that incorporate EBI and matrix training.

**Trial registration:**

CAAE#01454412.0.0000.5441 registered 01/29/2013.

## Background

Oral language skills are built when social environments jointly provide auditory experience and speech production, allowing children to acquire their first words to generate sentences (Fagan & Pisoni, 2010; Papalia & Olds, 2000). Listening and speaking skills depend on learning and are maintained by reinforcement contingencies in the verbal community (Greer & Ross, 2008; Skinner, 1957).

Sensorineural hearing loss that occurs before the acquisition of language, precluding sound detection below 70-dB, interferes with the typical development of oral language and hinders learning both listening and speaking skills (Fagan & Pisoni, 2010; Houston, Stewart, Moberly, Hollich, & Miyamoto, 2012). Professionals recommend rehabilitation and education approaches for populations with this limitation (Svirsky, Robbins, Kirk, Pisoni, & Miyamoto, 2000). Auditory speech-based approaches focus on auditory learning and oral language development, and residual hearing can be intensified with the use of electronic devices, such as hearing aids and cochlear implants (CIs) (Moog & Stein, 2008; Plant, 1997).

The present study assessed rehabilitation in children who used CIs to improve hearing function and establish oral language (Svirsky et al., 2000; Tobey, Geers, Brenner, Altuna, & Gabbert, 2003). Cochlear implants work through electrodes that are inserted in the cochlea where they electrically stimulate auditory nerve fibers. This stimulation provides auditory sensations of the frequency of speech and acoustic feedback for oral speech production (Spencer & Oleson, 2008). These conditions are important for learning that involves hearing and speech skills (Levine, Stother-Garcia, Golinkhoff, & Hirsh-Pasek, 2016; Svirsky et al., 2000), which can be operationalized as listening and speaking behaviors (Skinner, 1957).

Although CIs provide immediate auditory detection, more complex listening skills (e.g., auditory discrimination, recognition, and comprehension [Erber, 1982]) require learning (Fagan & Pisoni, 2010; Houston et al., 2012; Levine et al., 2016; Pisoni, 2000). Hollis, Fulton, and Larson (1986) verified the generality of Sidman’s (1971) equivalence model as a tool for teaching vocabulary in children with hearing loss. More recently, studies have extended the equivalence model (Sidman, 2000; Sidman & Tailby, 1982) to investigate and analyze meaning that is acquired from auditory stimuli in CI users (Almeida-Verdu et al., 2008; da Silva et al., 2006).

Equivalence relations are commonly established by the systematic training of two or more relations between stimuli or between stimuli and responses, with at least one common element (Sidman, 2000; Sidman & Tailby, 1982). This model provides an operational description of symbolic functioning. With regard to auditory stimuli, the equivalence model allows researchers to investigate whether subjects understand what they hear when other stimuli without physical similarity (e.g., text and pictures) become arbitrarily related and interchangeable under certain contexts. Symbolic relations exist if they attest to the formal properties of equivalence, which are symmetry (if A1rB1, then B1rA1), reflexivity (A1rA1 and B1rB1), and transitivity (if A1rB1 and A1rC1, then B1rC1) (Sidman & Tailby, 1982). The notation “r” means “is related to.”

Conditional relations among stimuli can be taught using matching-to-sample (MTS) procedures. In an MTS procedure, a stimulus serves as a sample and establishes the condition for another stimulus to exert a discriminative function and be selected among other available options (Mackay & Sidman, 1984; Sidman & Tailby, 1982). One example of MTS training would be that the object “ball” is correctly selected among all available options after presenting the auditory stimulus “ball.” Additionally, the printed word “ball” is correctly selected among all available options after presenting the auditory stimulus “ball.” In this example, all correct responses are reinforced. Equivalent relations between ball-object and ball-printed word will emerge after appropriate training, with the auditory stimulus “ball” serving as the common element for both trained relations. These derived relations confirm the transitive property of equivalence, demonstrate that the stimuli have become interchangeable or equivalent, and suggest that the learner exhibits auditory comprehension of the dictated word “ball” (Mackay & Sidman, 1984; Sidman, 1971; Sidman & Tailby, 1982).

Almeida-Verdu et al. (2008) and Battaglini, Almeida-Verdu, and Bevilacqua (2013) showed that children with CIs formed equivalence classes with auditory stimuli after stimulus-stimulus relation training using an MTS procedure. Other procedures were also used, such as fading and exclusion. Both studies found that the participants responded with the same name when equivalent pictures were presented, but their speech did not correspond to the dictated stimuli. These results showed that children with CIs produced inaccurate speech when tacting pictures (i.e., there was little correspondence when speech transcriptions were compared point-to-point with the target-written word with regard to conventions of the verbal community),^1^ even after the stimuli became equivalent. These results generally confirmed findings in the audiological literature that showed that this population has a rate of the acquisition of auditory skills that is close to their hearing peers, but the acquisition of oral production (or vocalization) does not follow that rate (Levine et al., 2016; Montag, AuBuchon, Pisoni, & Kronenberger, 2014; Pisoni, 2000). The time of auditory experience with the CI, the child’s age when CI surgery is performed, and language and hearing categories are variables that can predict language performance and affect this learning rate (Montag et al., 2014; Moog & Stein, 2008; Svirsky et al., 2000; Tobey et al., 2003). Tact is a verbal operant. Tacts that are evoked by nonverbal stimuli (e.g., objects, events, properties of objects or events, actions, and relations between objects and actions) can be vocal or signal topography and produce generalized conditioned reinforcement (Skinner, 1957; Sundberg, 2008). Language programs commonly observe tacts when the child name objects or pictures (Sundberg, 2008). Complex tacts are observed when several nonverbal component stimuli control verbal responding, generating sentences and other combinations among words (Skinner, 1957; Sundberg, 2008). In the present study, we adopted tacting scenes that referred to speech production using subject-verb-adjective-object combinations in response to pictures that showed scenes of people, actions, and objects (BD) (Golfeto & de Souza, 2015; Skinner, 1957; Sundberg, 2008).

Subsequent studies demonstrated the conditions under which speech accuracy in picture tacts can be achieved. Anastácio-Pessan, Almeida-Verdu, Bevilacqua, and de Souza (2015) and Lucchesi, Almeida-Verdu, Buffa, and Bevilacqua (2015) reported that children with CIs who were readers usually showed more accurate speech when reading text (i.e., textual behavior) than when tacting pictures.^2^ This discrepancy between expressive skills may be related to such variables as stimulus features and language experience (Jerger, Lai, & Marchman, 2002; Nation, Marshall, & Snowling, 2001). These studies also suggested the functional independence of verbal operants that are controlled by different stimuli (Guess, 1969; Skinner, 1957). Printed stimuli offer clues about which phonemes should be produced (i.e., grapheme-phoneme relation), whereas pictures do not provide any clues about what should be vocalized (Spencer & Oleson, 2008).

Equivalence-based instruction (EBI) can produce integration between verbal operants and derive one repertoire from another through the transfer of stimulus control (de Rose, de Souza, & Hanna, 1996; Mackay & Sidman, 1984; Sidman, 1986). Experimental studies have reported that populations with normal hearing first learn oral tacts (BD), and the accurate oral-textual operant (CD) is provided from equivalence relations that are formed (de Rose et al., 1996; Sidman, 1971). In an inverse route, children with CIs present improvements in oral-tact (BD) accuracy after EBI that includes oral-textual operants (CD) (Almeida-Verdu & Golfeto, 2016). In the study by Anastácio-Pessan et al. (2015), the participants were children with CIs who presented good oral-reading (CD) performance but inaccurate picture tacting (BD). Conditional relations between dictated words and pictures (AB) and between dictated words and printed words (AC word) were strengthened using an MTS procedure, including relations between dictated syllables and printed syllables (AC syllabic). The results showed that after strengthening equivalence relations (between dictated words, pictures, and printed words), accurate speech also started to occur under the presence of pictures. Analogously, Lucchesi et al. (2015) taught children with CIs who were not readers to relate pictures to dictated words (AB), printed words to dictated words (AC word), and printed syllables to dictated syllables (AC syllabic) using an MTS procedure. The training also included constructing printed words conditionally to dictated words using a constructed-response matching-to-sample (CRMTS) procedure. The participants learned to read and improved picture tacting.

The results of Anastácio-Pessan et al. (2015) and Lucchesi et al. (2015) demonstrated that children with CIs integrated reading and tacts in an equivalence-based network. Thus, the function of textual stimuli in speech was extended to pictures through transfer of the control of equivalence relations between pictures, dictated words, and printed words (Mackay & Sidman, 1984). These studies jointly suggested that children with CIs can improve picture tacting based on a previous reading repertoire using MTS training that strengthens stimulus-stimulus relations within an equivalence-based network and when minimal textual units are established.

Different procedures can establish conditional relations between auditory and printed stimuli and strengthen textual control. Anastácio-Pessan et al. (2015), for example, utilized an MTS procedure to teach conditional relations between dictated and printed stimuli (AC words and syllables) in children with CIs. A variant of the MTS is the CRMTS procedure, in which each component stimulus is selected in the appropriate order and conditioned to sample stimuli (Dube, McDonald, McIlvane, & Mackay, 1991). CRMTS training can produce the same conditional relations between auditory and textual stimuli, in addition to increasing control by each minimal unit (Calcagno, Dube, Galvão, & Sidman, 1994; Hanna, de Souza, de Rose, & Fonseca, 2004; Mackay & Sidman, 1984; Matos, Avanzi, & McIlvane, 2006). This procedure has been effective in teaching skills that involve reading and writing and recombining letters and syllables in several populations (Hanna et al., 2004; Stromer, Mackay, & Stoddard, 1992). This study used CRMTS of sentences in an EBI procedure in children with CIs.

Constructing sentences is a language milestone that depends on learning (Mackay, 2013; Papalia & Olds, 2000). It requires abstracting syntactic rules, categorizing words in classes, and establishing relations between words (or classes of words) that occupy a specific position in a sentence (Frampton, Wymer, Hansen, & Shillingsburg, 2016; Mackay, 2013). Order (or lexical responding) is at the base of such learning (Goldstein, Angelo, & Mousetis, 1987; Mackay, 2013; Skinner, 1957). Sentence productivity derives from recombinative generalization, in which the learner produces novel combinations by recombining words that were previously trained and arranged in order (Frampton et al., 2016; Goldstein, 1983b; Goldstein & Mousetis, 1989; Yamamoto & Miya, 1999). Some procedures can promote order-word relations and sentence productivity, such as matrix training (Axe & Sainato, 2010; Frampton et al., 2016; Goldstein, 1983a), CRMTS (Mackay, 2013), and matrix and CRMTS combined (Yamamoto & Miya, 1999).

Matrix training consists of distributing stimuli in a matrix so that intersections of rows and columns form combinations (Goldstein, 1983b). Some matrix combinations are taught, whereas others are only probed. Recombinative generalization is identified when the learner is able to produce untrained combinations of the matrix (Axe & Sainato, 2010; Goldstein, 1983a). With regard to receptive and expressive repertories, various populations have been successful in generating sentences with this training (Axe & Sainato, 2010; Ezell & Goldstein, 1989; Frampton et al., 2016; Goldstein & Brown, 1989; Goldstein & Mousetis, 1989; Mineo & Goldstein, 1990; Yamamoto & Miya, 1999). Arranging the matrix is critical for recombinative responding, and overlapping training can depend on unknown components and the learner’s baseline (Goldstein, 1983a, 1983b; Goldstein & Mousetis, 1989). Training with overlapping components improves discrimination and responding for each unit and effectively promotes vocabulary and the learning of syntactic rules simultaneously (Ezell & Goldstein, 1989; Goldstein, 1983a, 1983b; Goldstein & Brown, 1989; Golfeto & de Souza, 2015; Mineo & Goldstein, 1990). Non-overlapping training produces recombinative performance from the relation between components and is recommended when all of the components have been previously learned (Axe & Sainato, 2010; Frampton et al., 2016; Yamamoto & Miya, 1999).

The study by Golfeto and de Souza (2015) was one of the first to demonstrate the effects of matrix training on sentence learning in children with CIs. In their study, a 3 × 3 matrix was used in which three nouns were displayed in rows, and three verbs (in gerund) were displayed in columns. A constant object was added to the noun-verb combinations, thus generating nine sentences with a [subject]-[verb in gerund]-[object] structure. Children with CIs were taught to match video scenes to dictated sentences and produce echoic behavior for six sentences that overlapped verb-object or subject-object pairs. Tacting probes were conducted for all matrix sentences. These children learned the trained relations and increased the correct tacting of video scenes after echoic training, thus extending previous findings with words (Souza, Almeida-Verdu, & Bevilacqua, 2013). They were also able to tact novel scenes with diagonal sentences, demonstrating the potential of matrix training to generate sentences in children with CIs.

These recent findings encouraged further investigations of the conditions that are necessary to learn and produce sentences in children with CIs. Listening and echoic training produced tacting with sentences (Golfeto & de Souza, 2015). The present study employed another route by extending EBI to improve tacting from reading (Anastácio-Pessan et al., 2015; Lucchesi et al., 2015). We investigated whether children with CIs and readers present improvements in speech accuracy in tacting pictures of scenes using sentences with subject-verb-object combinations after EBI that included matching pictures to dictated sentences and constructing printed sentences under dictation. Given the potential of matrix training and that children with CIs were able to tact diagonal sentences from pair-component overlapping training (Golfeto & de Souza, 2015), we also assessed whether training diagonal sentences promotes recombinative performance on tacting with untrained sentences.

## Method

### Participants

The participants were three female children, 10–12 years of age, who were diagnosed with prelingual bilateral sensorineural deafness and used a unilateral cochlear implant (CI) and contralateral hearing aid. These children were patients at the Craniofacial Anomalies Rehabilitation Hospital in Bauru, Brazil, where they underwent CI implantation surgery. They periodically returned to the Audiological Research Center (CPA/HRAC) for follow-up and checkups of their CI’s function. They attended elementary education and received auditory-oral (re)habilitation by the hospital’s educational service. Consent forms were obtained from both the parents and the participants, and all ethical precautions followed the hospital’s protocols (CAAE 01454412.0.0000.5441).

Participants were recruited at CPA/HRAC after an evaluation by a speech therapist. None of the participants had a pre-study history of performing tasks that involved sentences as complex stimuli. They were evaluated in tasks that involved printed sentence reading and tacting pictures of scenes using the study stimuli. All of the participants met the inclusion criteria: <  50% speech accuracy in tacting pictures of scenes and > 70% accuracy in reading. Speech accuracy was measured by the percentage of point-to-point correspondence between speech that is produced by the child and speech conventions of the verbal community (Camarata, 1993).

The Peabody Picture Vocabulary Test Form A (PPVT-4) (Dunn & Dunn, 2007)^3^ and the Columbia (Alves & Duarte, 2001) test were administered individually and provided measures of receptive vocabulary and intellectual maturity, respectively. Hearing (Geers, 1994) and language (Robbins & Osberger, 1991) categories were assessed in speech therapy^4^ evaluations. Table 1 summarizes the characteristics of each participant.

**Table 1:** Characterization of participants by age, time of CI use, CI model, hearing categories (CatAud), language categories (CatLing), maturity index’s Columbia (considering level appropriate for chronological age), result’s PPVT (Peabody Picture Vocabulary Test-IV) and school grade

Participant	Age (years)	Time of CI use (years)	CI model	Cat Aud	Cat Ling	Maturity Index’s Columbia	PPVT results	School grade
LIV	11	6	Nucleus 24K CI24RST	6	4	High average	5:9	4 year
RAY	12	10	Nucleus 24K CI24RST	6	5	Boderline	6:9	5 year
LET	10	7	Hi Res 90K IC 1400-01	5	4	Average	5:6	3 year

All of the participants had receptive vocabulary scores that were lower than expected (Peabody Picture Vocabulary Test [PPVT] scores less than − 2 SD). Hearing categories of these children showed that they had the ability to recognize words by consonants (category 5) and in open set (category 6). With regard to expressive language, the participants were able to emit sentences (category 4) or had fluency in oral language (category 5).

### Settings and apparatus

The sessions were conducted at the cochlear implant service (CPA/HRAC) and at UNESP’s Laboratory of Learning, Development and Health, both within public universities in the state of São Paulo, Brazil. The data collection rooms had good lighting, were quiet, and were organized with tables, chairs, and toys.

A laptop computer that was equipped with external speakers and PROLER software, Version 6.4 (Assis & Santos, 2010) was used for data collection. The software presented the tasks, displayed programmed consequences, and recorded both stimulus selection and construction responses. The participants’ oral production was recorded with a Sony DCR-SR20 camcorder.

The computer program presented three types of trials. In stimulus selection trials, the sample stimulus was displayed on the center window, and three windows at the corners of the screen displayed comparison stimuli. The comparison stimuli were selected by clicking the computer mouse. In construction trials, the sample stimulus was displayed on a window on the top of the screen, and 12 windows on the bottom part of the screen presented the construction stimuli. The construction area had nine windows that were displayed between the sample and construction stimuli. Each construction stimulus was selected by clicking and dragging it to the construction area. When stimulus selection and construction trials had auditory samples, the program displayed a blue square in the center window. A click on the blue square simultaneously played the auditory stimuli on the speakers and displayed the comparisons or construction stimuli on the screen. In oral production trials, the program displayed a target stimulus in the center window, and the participant’s voice was captured by the camcorder.

### Experimental stimuli

**Fig. 1:**
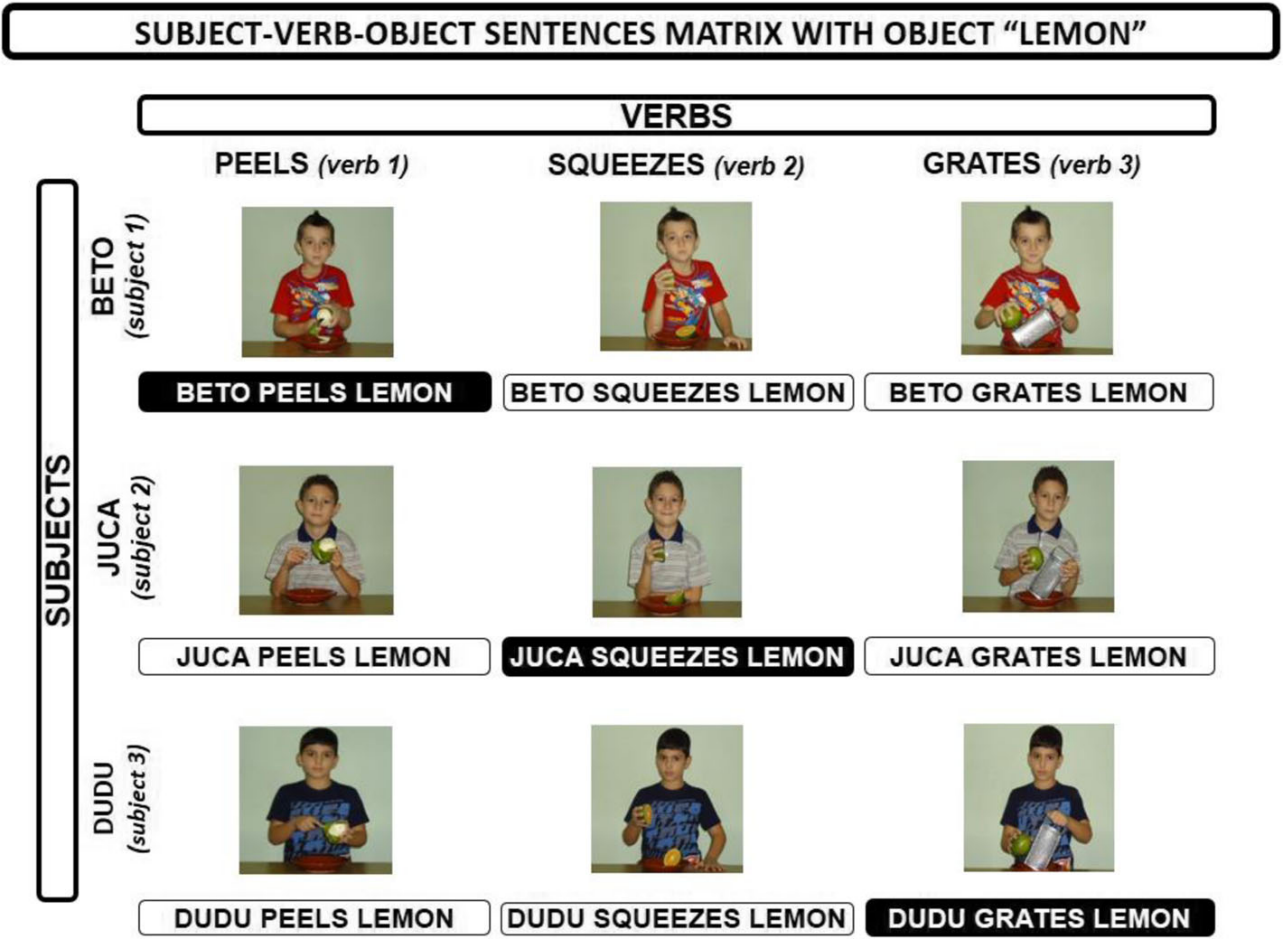
3 × 3 training matrix. The combination of the components in the rows (subjects), columns (verbs), and the constant object in the matrix generate nine subject-verb-object (S-V-O) sentences. The three diagonal sentences were directly taught (black box). The others six sentences in the matrix were tested (white box)

The present study adopted three-word sentences with a [subject]-[verb]-[object] (S-V-O) structure. Stimuli were planned using a 3 × 3 matrix. The subjects were arranged in rows. Verbs in the present tense were arranged in columns, and the object remained constant. The combination of the components in the rows, columns, and constant object in the matrix generated nine S-V-O sentences. Three diagonal sentences were taught. The other six sentences in the matrix were used in recombinative generalization probes. Figure 1 shows the distribution of linguistic stimuli in the matrix.

We used auditory and visual stimuli based on the matrix. Set A was composed of three sentences, “Beto peels lemon,” “Juca squeezes lemon,” and “Dudu grates lemon,” that were dictated in Portuguese (“Beto descasca limão,” “Juca espreme limão,” and “Dudu rala limão,” respectively). These were recorded with a female voice and played on the speakers. Set B included color digital images (500 × 500 pixels) that showed scenes of children who were performing actions that corresponded to the dictated sentences. Set C consisted of corresponding printed sentences that were typed in 65-point Arial font and were 3 × 3 cm.

### Procedure

#### Overview

Experimental sessions were conducted individually and lasted approximately 30 min. They were conducted once daily, with at least three sessions per week. In the first session, the researcher provided instructions and showed the participants how to operate the software. In the following sessions, the participants were seated in front of the computer screen. A researcher sat behind them and selected the blocks for each session. The participants performed the tasks in the software until the end of the block of trials. This was followed by 10 min of playtime. The participants received gifts (e.g., small toys, pencils, and storybooks) for their participation at the end of each session.

**Fig. 2:**
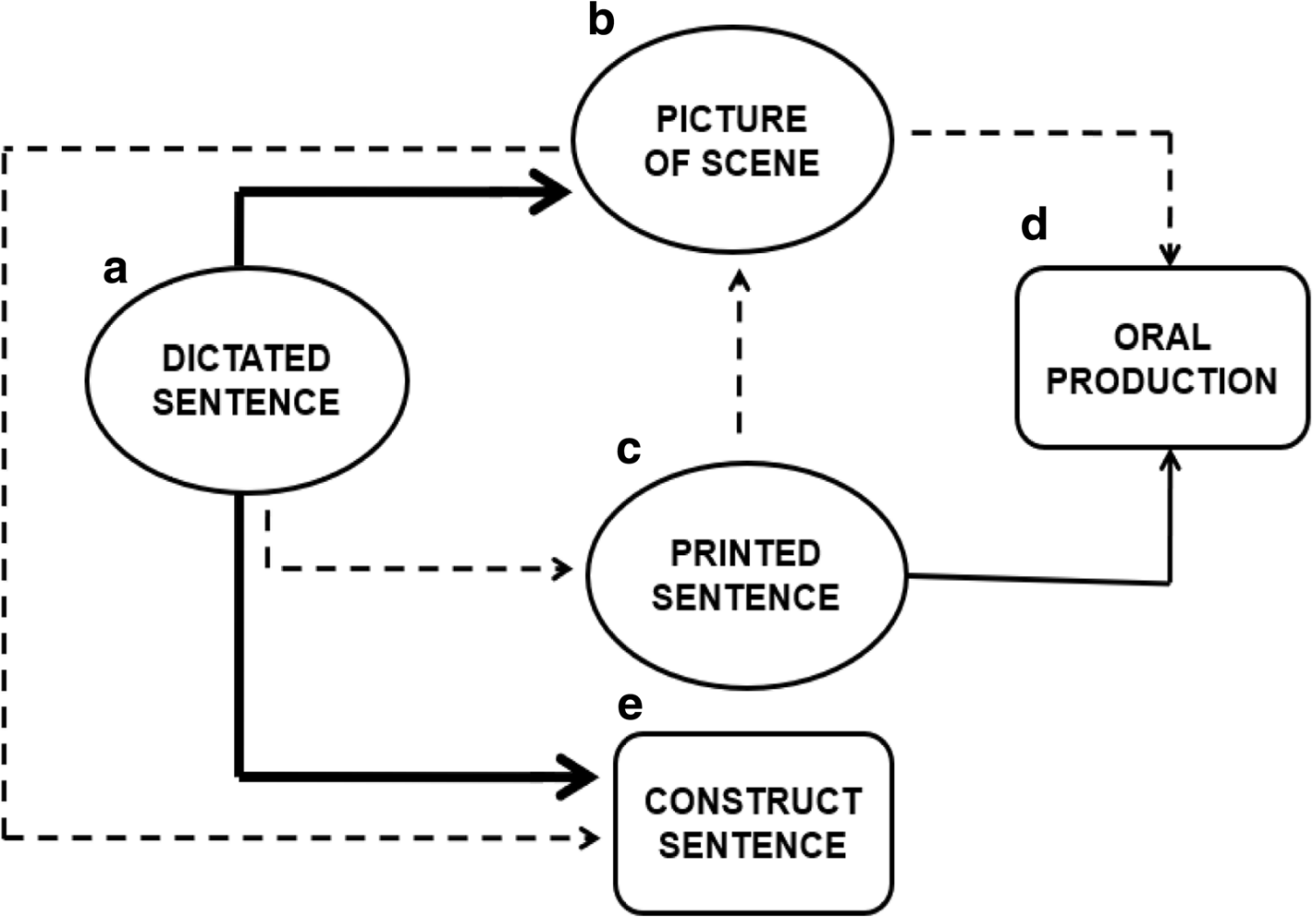
Equivalence relations network in present study. Adopted from Mackay (2013). Circles A, B, and C represent stimuli. Box D represents oral production by the participant. Box E represents word-by-word construction of the sentences. Arrows indicate relations and point from sample to comparison stimuli of matching-to-sample or constructed-response matching-to-sample tasks (AB, AC, CB, AE, and BE); arrows that indicate verbal operants point from antecedent stimuli to response (CD and BD). Solid arrows show relations already learned (CD). Heavy solid arrow shows trained relations (AB and AE). Broken arrows identify untrained relations

The study planned EBI. Figure 2 shows the trained (solid arrows) and tested (dashed arrows) relations in the equivalence network.

The procedure included pretests, training, posttests, and follow-up tests. All of the trials were discrete trials that included an instruction, a particular response, consequences (only for training conditions), and a 1-s intertrial interval (ITI). The positions and sequences of the comparison stimuli varied randomly within trials in all of the blocks. All of the tests were conducted without programmed consequences. Differential consequences for correct responses were only presented during the training phases. These consequences were animated GIF images and social compliments (e.g., “Very good”). Incorrect responses ended the trial with the presentation of a black screen for 3 s (timeout).

#### Pretest

Pretests ended after approximately 30 min and were conducted in a single session. Probes with three trained sentences (matrix diagonal) involved stimulus-stimulus and constructed response-based relations and verbal operants. Stimulus-stimulus relations were dictated sentence-picture (AB), dictated sentence-printed sentence (AC), and printed sentence-picture (CB). Constructed response-based relations were controlled by auditory stimuli (AE) and pictures (BE). Verbal operants involved speech production and included tacting pictures of scenes (BD) and reading (or textual, CD). The six recombined sentences were probed only for tacting (BD) and reading (CD). Each trial presented one relation for each sentence, for a total of 33 trials in the pretest. The trials were presented in blocks according to the requested response (stimulus selection, construction, or oral production) and had no programmed consequences.

Stimulus-stimulus relations were probed in a block with nine MTS trials, three dictated sentence-picture (AB) relations, three dictated sentence-printed sentence (AC) relations, and three printed sentence-picture (CB) relations. Every MTS trial simultaneously presented the sample and the comparisons, which were available until the participants selected one of the comparison stimuli. In auditory-visual trials (AB and AC relations), the participants had to click on the blue square, and the auditory sample was repeated every 3 s until selection of the comparison stimulus. These responses were followed by removal of the stimuli and presentation of the ITI.

Construction-based trials involved constructing printed sentences that were conditioned to the auditory (AE) and visual (BE) samples and were probed in a block with six CRMTS trials. The CRMTS trials simultaneously presented a sample stimulus and a pool of words from which to choose. The pool only contained 3 words that matched to the sample and were randomly presented within 12 available cells. Auditory-visual (AE) trials required a click on a blue square that displayed the word pool and played the auditory sample every 3 s. The participants were required to click on the words in order, which moved them to the construction area, from left to right. The trials ended with the selection of the last word and were followed by the removal of stimuli from the screen and presentation of the ITI.

The relations that required speech production were included in 18-trial blocks. The trials probed the tacting and reading of all matrix sentences (i.e., three trained sentences [matrix diagonal] and six recombined sentences). Each trial evaluated one verbal response of tacting pictures of scenes (BD) or reading (oral textual, CD) one sentence. The trials began with a blue square at the center of the screen, and the participant had to click on it with the mouse. This response was simultaneously followed by both presentation of the target stimulus in the center window and by an auditory prompt that was presented through the speakers. Tacting (BD) trials displayed a picture of a scene with the prompt, “What is happening?” (in Portuguese). Reading (CD) trials displayed a printed sentence and prompted, “What is written?” (in Portuguese). The vocal response was recorded by the camcorder, and the trial ended when the participant clicked on the target stimulus.

#### AB training

Conditional discriminations between dictated sentences and pictures of scenes (AB relation) were trained by a trial-and-error procedure with only three diagonal sentences. Every training block had nine MTS trials, three for each AB relation (A1B1, A2B2, and A3B3). The position of successive samples and simultaneous comparisons alternated within blocks. Trials began with display of the blue square in the center window. One click simultaneously enabled both the dictated sentence through the speakers and the presentation of three pictures at each corner of the screen. The auditory sample was repeated every 3 s until comparison stimuli were selected. The participants responded correctly when they selected a comparison stimulus that was arbitrarily matched to the sample. Correct and incorrect responses were followed by differential consequences and the ITI. The learning criterion was 100% correct responses in the AB training block. If the participants did not meet this criterion, then the block was repeated twice in the same session. The session ended when the participant made an error in the second repetition of the training block.

#### AE training

Printed sentence construction that was controlled by dictated sentences (AE relation) was also trained using a trial-and-error procedure and only involved three diagonal sentences. Each block had three CRMTS trials and presented one target relation (A1E1, A2E2, or A3E3) at a time. The trials initially displayed a blue square in the center window, and clicks on it were followed by simultaneous display of the dictated sentence and a word pool at the bottom of the screen. The word pool included three words from the target sentence, and the positions alternated for each trial. Auditory samples were repeated every 3 s until the participants finished the construction. The correct constructed response consisted of selecting each word in the appropriate order, conditional to the dictated sentence. Differential consequences were provided for every correct and incorrect response.

AE training began with a block of trials for the sentence, “Beto peels lemon.” (A1E1). The learning criterion was 100% correct responses in each AE block. If the participants met the learning criterion in this block, then they proceeded to the AE block with the sentence, “Juca squeezes lemon.” (A2E2). The final AE block consisted of the sentence, “Dudu grates lemon.” (A3E3). If the participants did not succeed, then the same block was repeated no more than twice in the same session. If the participants produced one error in the second repetition, then the session ended, and training was resumed in the next session.

After training each AE relation, the participants were trained with all three relations (A1E1, A2E2, and A3E3) in a mixed block of trials. This block was composed of nine CRMTS trials, with three trials per construction-based relation. Samples alternated in successive trials, and the word position changed randomly in every trial. The learning criterion was also 100% correct constructed responses. If the participant did not meet this criterion, then a repetition of each trained AE sentence was conducted with the same criterion before proceeding to the integral baseline training.

#### Posttests and follow-up tests

Posttests and follow-up tests lasted approximately 30 min and were conducted in separate sessions. The tests were conducted without programmed consequences. In these steps, all stimulus-stimulus, construction-based, and speech production relations that were presented in the pretests were probed again. The trial blocks and procedures were the same as in the pretest.

Posttests were conducted after training (or after repeating the training block with AB and AE trials) and evaluated the effects of training on the trained and derived relations and speech accuracy in tacting using trained and recombined sentences. Follow-up tests occurred after 1 week and again 1 month after the participants ended the study and were conducted to probe learning retention.

### Data analyses

PROLER software recorded responses in the blocks of trials and generated reports of the participants’ performance. Performance in the MTS and CRMTS trials were analyzed individually as the percentage of correct responses for each relation and block.

Speech production responses in tacting (BD) and reading (CD) were videotaped and submitted for speech transcription. Transcriptions were scored by checking for point-to-point correspondence between the children’s speech and the target phoneme according to linguistic conventions of Brazilian Portuguese. Speech accuracy was individually analyzed in terms of percentage and calculated as the ratio between the number of correctly emitted phonemes and the total number of phonemes of each target sentence (Camarata, 1993). For example, saying “Dudu grakes lemon” (14 correct phonemes) for the target sentence, “Dudu grates lemon” (15 phonemes in total), produced a ratio of 14/15, yielding a percentage of speech accuracy of 93.33%.

### Interobserver agreement

Interobserver agreement was obtained for oral responses in tacting and reading probes. The researcher and a second observer separately watched the videos and transcribed the speech of each participant independently. Interobserver agreement was computed using the following formula (Kazdin, 1982): total number of agreements divided by agreements plus disagreements, multiplied by 100 (number of agreements / [number of agreements + number of disagreements] × 100). The interobserver agreement for each sentence was ≥ 93.89%.

### Design

The study employed a multiple baseline design across participants (Kazdin, 1982). Speech accuracy in tacting pictures of scenes was simultaneously evaluated across participants. Training was introduced in one participant while baseline continued for the other participants. The next participant then began training when the previous participant presented stable performance during training. In the present study, the multiple baseline design allowed experimental control and assessment of the effects of EBI (independent variable) on speech accuracy in tacting scenes (dependent variable), with three participants and at three different time points.

## Results

The three participants completed all phases of the study. The total number of sessions varied between participants, and the interval between the last pretest and last posttest was an average of 30 days, not including follow-up tests.

### Probes of both stimulus-stimulus and construction relations

Table 2 shows the results of the participants in the pretests and last posttest. The probes included stimulus selection and construction blocks with the same relations (AB, AC, CB, AE, BE) in the equivalence network for trained sentences.

**Table 2:** Performance of participants in stimulus selection (CB, AC, and AB) and sentences construction (AE and BE) during the pretests and posttest probes for trained sentences

	Stimulus selection tasks						Sentences construction tasks			
	Printed sentence–picture of scene (CB)		Dictated sentence–printed sentence (AC)		Dictated sentence–picture of scene (AB)		Dictated sentence–printed sentence construction (AE)		Picture of scene–printed sentence construction (BE)	
	Pre	Post^a^	Pre	Post^a^	Pre	Post^a^	Pre	Post^a^	Pre	Post^a^
LIV	33.3		33.3		33.3		100		100	
	66.7	100	100	100	66.7	100	100	100	100	100
RAY	0		66.7		33.3		100		100	
	33.3		100		66.7		100		100	
	33.3	100	100	100	33.3	100	100	100	100	100
LET	33.3		0		0		100		100	
	33.3		66.7		0		100		66.7	
	33.3		66.7		33.3		100		100	
	0	100	66.7	100	33.3	100	100	100	100	100

^a^The posttest data refer to last exposure

In the pretests, all of the participants presented well-established construction responding from the beginning of the study and had practically 100% correct responses for printed sentences for auditory (AE) and visual (BE) samples. High variability in performance among the participants was found in the MTS procedure. Tasks that required auditory conditional control produced more correct selections of printed sentences (AC) compared with pictures of scenes (AB). All of the participants, except LET, gradually increased their number of correct responses when matching printed sentences to dictated sentences (AC), obtaining 100% correct responses in the last pretest. LET improved performance from 0% correct in the first test to 66% correct in the following pretests. In tasks that required matching pictures to dictated sentences (AB), the participants presented variable performance in the pretests, and all of them concluded the last pretest with 33.3% correct responses. Variability was also verified for the CB relation, and the participants’ percentage of correct responses was between 0 and 66.67% when matching pictures to printed sentences, with more likely performance of 33.33%.

In the posttests, all of the participants maintained 100% accuracy for the construction of printed sentences under auditory and visual control (AE and BE, respectively) and matching printed sentences to dictated sentences (AC). While the other participants were 100% accurate in the AC relation during the pretests, LET only reached this performance after training. Auditory control over picture selection was obtained after training, and all the participants had 100% correct responses when matching pictures to dictated sentences (AB). After training, the CB relation was derived, and all of the participants had 100% accuracy when matching pictures to printed sentences.

### AB and AE training

During training, all of the participants met the 100% accuracy criterion when matching pictures to dictated sentences (AB) and constructed sentences under dictation (AE). Each participant met the learning criterion at a different time. The amount of exposure to training blocks that were needed to meet the criterion varied (Table 3).

**Table 3:** Number of exposures to training blocks to meet the learning criterion

	Training				
	Matching pictures of scenes to dictated sentences (AB)	Printed sentence construction under dictation (AE)			
	(A1B1, A2B2, A3B3)	A1E1	A2E2	A3E3	AE (A1E1, A2E2, A3E3)
LIV	6	1	1	1	1
RAY	12	1	1	3	1
LET	8	1	1	1	1
Average	8.66	1	1	2	1

All of the participants had more difficulty in tasks that related auditory stimuli and pictures and repeated training blocks that matched pictures to dictated sentences (AB). The range of exposure that was necessary to meet the learning criterion in AB training was 6–12 exposures (average 8.66). In AE training blocks, all of the participants, except RAY, met the learning criterion after one exposure. RAY required two repetitions to correctly construct the printed sentence when the sentence, “Dudu grates lemon,” was dictated.

### Probes of reading and tacting pictures of scenes

**Fig. 3:**
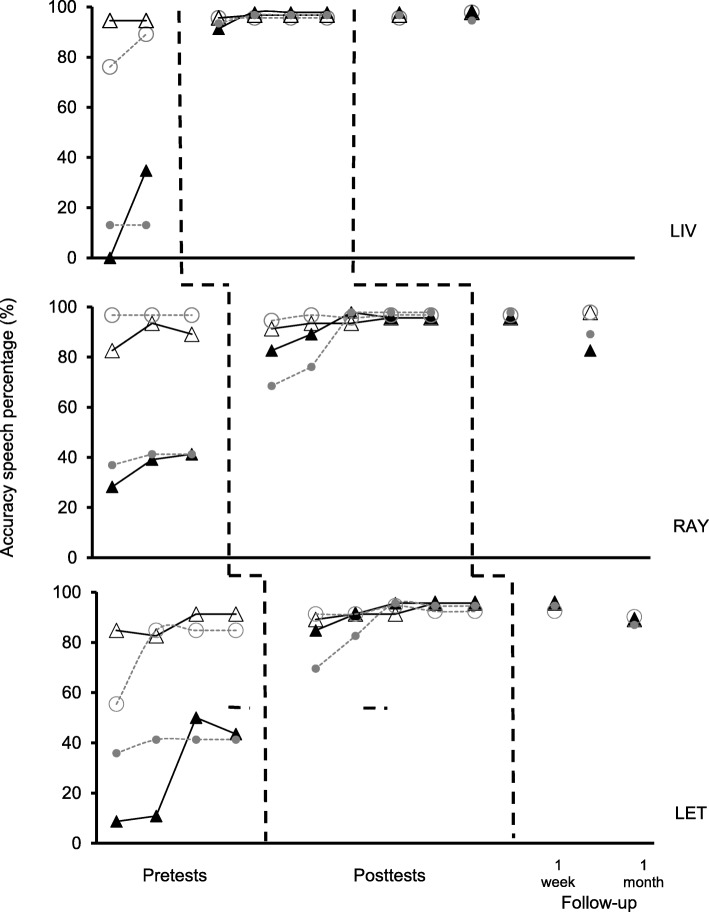
Accuracy speech percentage in reading (CD) and tacting pictures of scenes (BD) probes. The sentences directly taught (black triangles with solid line) refers diagonal of matrix and recombined sentences (gray circles with dashed line) to others sentences in the matrix. The open markers indicate reading performances, while closed markers indicate tacting pictures of scenes performances

Figure 3 shows the participants’ performance on reading (CD) and tacting (BD) probes. Speech accuracy in reading (open markers) and tacting pictures of scenes (closed markers) are shown for trained (black triangles with solid line) and recombined (gray circles with dashed line) sentences according to a multiple baseline design across participants (dashed line).

Speech accuracy was generally well established and maintained at a high level under textual control. All of the participants showed stable performance above 70% for all reading (CD) probes. Accuracy was low when the picture was shown (BD), and the participants produced phonetic distortion, omission, and substitutions in the pretests, with performance below 50%. Thus, there was a discrepancy between reading (CD) and tacting pictures of scenes (BD) before training. After AB and AE relations were trained, the participants increased their speech accuracy in tacting pictures of scenes (BD), reaching performance that was similar to reading (CD; > 70%). Thus, tacting (BD) performance approximated reading (CD) performance after training.

With regard to trained sentences (matrix diagonal), all of the participants produced more accurate speech for printed sentences than for pictures of scenes, which were maintained below 50% and above 70%, respectively, from the first to the last pretest. Performance changed little in successive pretests, and the discrepancy between reading (CD) and tacting (BD) was always above 60% on average. Reading (CD) performance was 86.71% accurate on average, and all of the participants increased their speech accuracy by approximately 8.7% from the first to the last pretest. Tacting pictures of scenes (BD) had greater variability, with an average of 28.51% and range of 40%, and the increase in speech accuracy from the first to the last pretest was of 34% for LIV and LET and 13.04% for RAY.

Reading (CD) performance in the posttests was 93.94% accurate on average, with a range of 6.52%. From the last pretest to the last posttest, the participants presented a 5.8% increase in reading (CD) with the trained sentences and reached 95.65% accuracy in the last probe. Tacting (BD) in the posttests was 93.48% accurate, with a range of 15.22%. All of the participants vocalized the trained sentences when tacting pictures of scenes, and speech accuracy was above 80% in the probes, with an increase of 56.52% on average from the last pretest to the last posttest. Thus, tacting scenes only reached reading levels for all of the participants after training (average of 93.94% in CD and 93.48% in BD).

In the 1-week follow-up probe, the participants maintained the same performance for reading (CD) and (BD) tacting trained sentences that had been shown in the last posttest. At the 1-month follow-up, the participants’ reading (CD) performance was unchanged or increased moderately (less than 10%), and tacting (BD) performance decreased slightly (less than 8%) but never returned to baseline levels.

The results with trained sentences (matrix diagonal) were replicated with sentences that had not been directly trained. Reading (CD) performance (average = 87.68%; range = 41.31%) was higher than tacting (BD) (average = 33.93%; range = 28.26%) during the pretests, and the discrepancy between reading (CD) and tacting (BD) was maintained at the same level as the trained sentences (above 60% discrepancy). After training, tacting (BD) (average = 89.91%; range = 29.35%) also approximated reading (CD) performance (average = 94.88%; range = 5.44%), as had been observed with the trained sentences. Reading (CD) and tacting (BD) performance was maintained in the 1-week follow-up test. At the 1-month follow-up, reading (CD) performance was the same or higher (less than 11%), and tacting (BD) performance decreased (less than 10%) but did not return to baseline, which was 33.93% on average.

## Discussion

The present study investigated whether children with CIs who were readers improve their speech accuracy when tacting pictures of scenes (BD) after EBI that involved matching pictures of scenes to dictated sentences (AB) and constructing printed sentences after dictation (AE). We also used matrix training to evaluate whether teaching verbal relations with diagonal sentences would promote recombinative generalization, especially in tacting pictures with untrained sentences. Overall, our hypothesis was confirmed, and all of the participants learned the trained relations, presented the derived relations, and demonstrated equivalence relations between dictated sentences, pictures of scenes, and printed sentences. After EBI, the children also increased their speech accuracy in tacting pictures of scenes (BD relations) for trained sentences and were able to recombine sentence components and accurately tact pictures with untrained sentences.

All of the participants met the inclusion criterion by demonstrating better speech accuracy when reading text (CD) than when tacting pictures (BD) in the pretests. However, the initial discrepancy between tacting (BD) and reading (CD) requires further explanation. One hypothesis is that this performance reflected an experimental artifact. Our participants were readers, and graphemes functioned as cues for accurate pronunciation in reading (CD). In contrast, pictures of scenes do not provide sufficient clues for tacting (BD) with target sentences. Such characteristics as photo format, the topography of actions, context of the scenes, static motion, and other unknown elements may interfere with tacting pictures (Nation et al., 2001) and may have hindered the participants’ performance in the present study. Future studies should enhance the pictures by using dynamic motion, making the topography of actions clearer in the scenes, and reducing unknown components, which may ultimately minimize possible artifacts of tacting pictures of scenes.

However, tacting scenes can occur independently of artifacts. Children with language experience may use strategies to tact unknown pictures, such as describing scenes, using pronouns, and reporting the semantic context (Jerger et al., 2002; Nation et al., 2001). None of the participants in the present study emitted these types of responses. Given that language experience is an important variable that affects tacting pictures, objects, and events, the children in the present study began auditory-verbal learning after CI implantation surgery and had recent contact with oral language compared with their hearing peers, which may explain why they tacted so little and did not use the aforementioned language strategies.

In the present study, the speech transcriptions clarified this issue, showing that the participants’ speech accuracy was handicapped. The children tacted pictures of scenes with little (if any) point-to-point correspondence with the target sentences. Such sentences as “Beto descasca limão” were spoken as “tatando faca imao” (inaccurate verb and object), “uaistemeimão” (other word in place of verb and object with little correspondence), and “cota limão” (verb with phonetic omission). Some phonetics or phonological inventories (Yavas, Hernandorena, & Lamprecht, 2001) would provide additional data about the participants’ speech performance and may be implemented in future studies. Indeed, our hypothesis is that verbal topography may have little correspondence with the target sentence because of differences in stimulus control (written sentences and pictures of scenes) and some clusters of consonants.

These results are consistent with previous studies of the speech production of children with CIs and confirm poorer levels of speech intelligibility when they name pictures (Montag et al., 2014; Tobey et al., 2003). Phonetic omissions and distortions (i.e., the omission of “r” in “corta” for RAY) and substitutions by another word (i.e., LET said “uaiste” instead of “descasca”) were more frequent mistakes when they tacted pictures. Such occurrences replicate audiological findings about this characteristic of spoken language in children with CIs (Montag et al., 2014; Spencer & Oleson, 2008; Svirsky et al., 2000).

Considering that the difference in performance between reading (CD) and tacting pictures of scenes (BD) could derive from stimulus control, oral production in the presence of one stimulus can occur regardless of situations that require other stimuli because verbal responding depends on the specific relation with the stimulus that preceded it (Greer & Ross, 2008; Skinner, 1957). Our pretest data corroborate previous studies that showed functional independence between verbal operants (Guess, 1969; Skinner, 1957) and suggest that speaking correctly from printed sentences does not guarantee that children with CIs will accurately tact pictures of scenes. The present study extends to sentences what previous studies had already shown with single words (Anastácio-Pessan et al., 2015; Lucchesi et al., 2015). These findings encourage further investigations of the conditions that promote interdependence between verbal repertories in children with CIs, such as rotation among operants (Greer & Ross, 2008) and equivalence-based instruction (Mackay & Sidman, 1984; Sidman, 1986).

The present study promotes equivalence relations as a route to integrate reading (CD) and tacting (BD), thus improving speech accuracy in tacting pictures of scenes (BD) using sentences in children with CIs. After training that matched pictures to dictated sentences and constructed printed sentences under control of the same dictated sentences (AB and AE, respectively), the children formed equivalence relations between dictated sentences, pictures of scenes, and printed sentences. They also tacted pictures with greater point-to-point correspondence to target sentences, improving speech accuracy to reading levels. The results of the posttests indicated functional interdependence between reading (CD) and tacting (BD). These results also corroborate previous findings about the ability of equivalence relations to produce interrelations between verbal operants and establish one repertoire from another (de Rose et al., 1996; Hollis et al., 1986; Mackay & Sidman, 1984; Sidman, 1971; Sidman, 1986). Some studies in the area of equivalence used oral tacts (BD) to provide oral-textual (CD) responses (de Rose et al., 1996), whereas the present study and other studies that evaluated children with CIs used an inverse route and improved tacting pictures (BD) from previously established reading (CD) skills (Almeida-Verdu & Golfeto, 2016).

Equivalence classes allowed extending discriminative control that printed sentences had on speech accuracy to other equivalent stimuli, such as pictures of scenes. The transfer of control from equivalence relations was found in this study (Mackay & Sidman, 1984; Sidman, 1986) and replicated studies that showed that speech accuracy in children with CIs was transferred from text to pictures after equivalence formation using single words (Anastácio-Pessan et al., 2015; Lucchesi et al., 2015). Our results replicate previous findings (Anastácio-Pessan et al., 2015; Lucchesi et al., 2015) and expand such findings to units that are larger than words, such as sentences. Considering potential contributions to clinical practice, these results also encourage future investigations of the detailed effects of EBI on the acquisition of or changes in the production of speech sounds at the sentence level.

With regard to the interdisciplinary nature of this interface, some previous findings suggest that accurate tacting is related to the time of auditory experience with CIs, age when CI implantation surgery is performed, and language and hearing categories (Montag et al., 2014; Moog & Stein, 2008). Relations between these variables were not clearly identified in the present study. Participant RAY had the longest auditory experience (10 years), earliest time of CI implantation (2 years old), and better language and hearing categories (6 and 5, respectively) but showed learning and tacting improvements that were similar to the other participants. Although there was a difference between the participants, our results suggest that gains in speaking and listening depend on the integration of these variables in a multifactorial process (Montag et al., 2014; Svirsky et al., 2000; Tobey et al., 2003). Within the scope of this study, the teaching procedure was a determinant of improvements in tacting. Future research should adopt specific inclusion criteria and select a more homogeneous sample to verify the effects of these variables.

Training in matching pictures to dictated sentences (AB) required a greater number of repetitions for all of the children, with an average of 8.66 exposures to meet the learning criterion. These results differ from the literature on auditory-visual relational learning in children with CIs (Almeida-Verdu et al., 2008; Anastácio-Pessan et al., 2015; Battaglini et al., 2013). Some hypotheses can be formulated based on these findings.

The complexity of auditory stimuli could have interfered with the participants’ auditory-visual MTS performance. Dictated sentences are stimuli that last longer, have more phonemes, require more segmentation, and can include lexical difficulty (Spencer & Oleson, 2008). Stimulus complexity can make the task harder by demanding both previous auditory skills and more time for children with CIs to learn to recognize larger units (Svirsky et al., 2000). Some rehabilitation approaches suggest teaching the auditory recognition of small units before advancing to sentences (Erber, 1982; Plant, 1997). The auditory recognition of sentences by children with CIs requires further research to probe the effects of experimental manipulations on stimulus complexity (e.g., by adding articles). Beyond that, the phonological balance must be adjusted experimentally to control some linguistic difficulties that are encountered with sentences (Spencer & Oleson, 2008). In the present study, the participants may have presented difficulties recognizing the verbs *descasca* and *espreme* because they were unfamiliar and had auditory requirements to discriminate these consonant clusters. Moreover, our participants also presented inaccurate speech with these verbs during tacting pictures in the pretests. This result corroborates previous findings that showed that speech accuracy in children with CIs may be related to linguistic characteristics of the words and increasing difficulty in pronunciation, such as with “sc” (*descasca*) and “pr” (*espreme*) in the present study (Spencer & Oleson, 2008).

Repetitions of auditory-visual MTS training can also be related to procedural aspects. The present study employed a trial-and-error procedure that could entail more repetitions (Ferrari, de Rose, & McIlvane, 1993). Most studies with children with CIs used training procedures that consisted of shaping stimulus control to produce learning with few errors (Almeida-Verdu et al., 2008; Anastácio-Pessan et al., 2015; Battaglini et al., 2013). Future research should employ errorless training procedures (e.g., fading [Terrace, 1963] and teaching by exclusion [Dixon, 1977]) and probes and compare the effects of AB training on repetition and determine whether auditory-visual conditional learning with sentences is faster under such conditions.

Conditional relations between auditory and textual stimuli and their minimal units can be produced by several procedures (Mackay & Sidman, 1984; Matos et al., 2006). In the study by Anastácio-Pessan et al. (2015), for example, children with CIs learned conditional discriminations between dictated and printed words or syllables (AC-word or AC-syllabic) through an MTS procedure. We used CRMTS to teach conditional relations among the components of dictated and printed sentences and to maximize the chances of control by minimal units (Dube et al., 1991; Hanna et al., 2004). Positive results in AE training showed that CRMTS produced auditory-textual conditional relations and favored control by means of each sentence unit, as suggested by Mackay and Sidman (1984) and Matos et al. (2006). These findings can be incorporated into future research and the rehabilitation of children with CIs and confirm that CRMTS is useful for teaching relations between auditory and textual stimuli.

Accurate performance in AE training can also indicate that our participants formed (or strengthened) word-order relations. Samples were dictated sentences with the same S-V-O structure, and only the target words were presented for sentence construction. Thus, control by multiple factors may have operated on constructed responding. The conditional function of dictated sentences may have indeed controlled the selection of each word and established control by minimal units. The regular S-V-O order in the samples and the display of only target printed words may have functioned as cues for responding that was controlled by position or order, especially for children who already knew these syntactic rules. The ordinal relations in the present study are consistent with previous studies that indicated that the ordinal arrangement between stimuli might be a condition that favors the production and recombination of sentences (Mackay, 2013; Yamamoto & Miya, 1999). These data encourage future studies to control these variables and guarantee the topographical coherence of stimulus control (Matos et al., 2006). Joint control by the sample and the order of stimuli may be beneficial for sentence learning but needs to be verified experimentally so that knowledge about isolated and combined effects may serve as a basis for making decisions when teaching in applied contexts.

Positive posttests of BE and CB relations indicated that the participants formed equivalence relations among dictated sentences, pictures of scenes, and printed sentences (ABC). Constructing printed sentences in response to pictures (BE) confirmed the transitive property (Mackay & Sidman, 1984; Sidman & Tailby, 1982). The results of BE relations, together with accurate performance in the posttests that required matching printed sentences to pictures (CB), provided evidence that (i) relations between auditory and printed stimuli can be established by anagram tasks, (ii) stimuli that are produced using constructed responses are included in the equivalence class, and (iii) constructed responses integrate the equivalence-based network, together with reading and writing (Calcagno et al., 1994; Sidman, 2000; Stromer et al., 1992; Stromer & Mackay, 1992; Yamamoto & Miya, 1999).

The present study generally adds to Golfeto and de Souza’s (2015) operant research on sentence learning in children with CIs. Golfeto and de Souza (2015) adopted listening and echoic training to produce tact operants, and their study extended to sentences previous conclusions with regard to the effects of echoic training on tacts with words (Souza et al., 2013). The present study employed EBI to improve tacting pictures of scenes in children with CIs who were readers, which expanded to sentences the data that were reported by Anastácio-Pessan et al. (2015) and Lucchesi et al. (2015) with single words. Both routes can be useful to teach sentences to children with CIs. In initial stages of language acquisition, these children may require more training of rudimentary skills (e.g., listening and echoing) for tacting (Fagan & Pisoni, 2010), whereas children who already read can tact more accurately by learning equivalence between auditory, printed, and pictorial stimuli and transfer the control of speech from text to pictures (Almeida-Verdu & Golfeto, 2016).

Sentence productivity is a behavioral language process (Mackay, 2013; Skinner, 1957) that was demonstrated experimentally in the present study using matrix training (Goldstein, 1983a, 1983b; Goldstein et al., 1987; Goldstein & Mousetis, 1989; Mackay, 2013). Our participants had high performance in generalization probes after training. These findings converge with the literature that shows that matrix training can promote recombinative generalization within sentences for expressive and receptive repertories in different populations with normal hearing (Frampton et al., 2016; Goldstein et al., 1987; Yamamoto & Miya, 1999). These results replicate Golfeto and de’s Souza (2015) findings on recombinative generalization in children with CIs using a matrix and allows further discussion of the arrangement of matrix training.

Overlapping components can be a critical variable in matrix training (Goldstein, 1983a, 1983b; Goldstein & Mousetis, 1989). The present study and the study by Golfeto and de Souza (2015) used matrix training with different arrangements in children with CIs. The study by Golfeto and de Souza (2015) trained relations to sentences with two overlapping components (subject or verb plus a constant object), whereas the present study manipulated this variable and verified the effects of diagonal training that involved sentences with an overlapping object and non-overlapping subjects and verbs. Both studies confirmed previous findings that recombinative performance can be obtained with matrix training both with one overlap (Ezell & Goldstein, 1989; Mineo & Goldstein, 1990) or several overlapping components (Goldstein, 1983a, 1983b; Goldstein & Brown, 1989). Diagonal training appeared to be sufficient for the children with CIs to generate novel tacts, but this conclusion requires more empirical support and the control of variables, such as familiar or known stimuli and previous lexical skills (Goldstein, 1983b; Yamamoto & Miya, 1999). No-overlap training has also been effective in populations with minimum verbal repertoires (Axe & Sainato, 2010; Frampton et al., 2016; Goldstein & Mousetis, 1989; Yamamoto & Miya, 1999) and may be experimentally probed in children with CIs. Our results suggest that future research can maximize recombinative generalization by arranging matrices, such as by training with three-dimensional matrices. Experimenters could then teach three diagonal sentences and generate 24 of 27 sentences in a three-dimensional matrix, in contrast to the 6 of 9 sentences in a two-dimensional matrix.

Ordinal relations between components are at the basis of sentence learning and productivity (Frampton et al., 2016; Mackay, 2013; Skinner, 1957). Syntactic rules can be abstracted when learners categorize words and establish relations between words that occupy a specific position (Frampton et al., 2016; Goldstein et al., 1987; Mackay, 2013), which can be taught using a matrix (Frampton et al., 2016; Goldstein et al., 1987; Golfeto & de Souza, 2015) and CRMTS (Mackay, 2013). The present study combined matrix and CRMTS training. The findings support the hypothesis that these procedures together may have increased control by the ordinal position of words within a sentence, replicating with children with CIs Yamamoto and Miya’s (1999) findings using the same procedures. The greater control of order may be useful for the abstraction of relations between components and the formation of ordinal classes (Mackay, 2013) in children with CIs. However, this must be investigated empirically so that knowledge about isolated and combined effects can help define a sentence curriculum for this population, thus increasing the social validity of this work by elucidating relationships between literacy and generalized reading (Baer, Wolf, & Risley, 1968).

The present study employed a multiple-baseline design across participants, which had both advantages and limitations. Repeated measures of tacting pictures of scenes (dependent variable) allowed us to monitor the baseline and demonstrated that all of the participants had variable performance in tacting in the pretests. The data indicate instability of the dependent variable, which compromised experimental control. More stable baselines were noticed in other studies with children with minimal verbal repertoires (Frampton et al., 2016). However, children with CIs differed from these populations. The dependent variable presented some variations, with a tendency toward improvement in tacting pictures, objects, and events (Anastácio-Pessan et al., 2015; Golfeto & de Souza, 2015; Lucchesi et al., 2015). In the present study, the variability of tacting pictures was lower than 40% in the pretests for all of the children, which can be individually attributed to the absence of intellectual deficits and opportunities to learn language from auditory-oral rehabilitation, formal education, and incidental auditory experience. The experimental design also had advantages by controlling those intervenient variables. The first participant tacted 80% of pictures accurately after EBI, whereas the other two participants continued to perform at baseline levels and maintained tacting picture accuracy below 50% because none of the participants were exposed to an intervention. Moreover, these effects of EBI on speech accuracy in tacting pictures of scenes were replicated in other participants. Thus, the internal validity of the study was strengthened by demonstrating the effects of the independent variable on the dependent variables, which was replicated for all of the participants. Future studies should seek to replicate these findings with more participants in other laboratories and by manipulating other parameters (e.g., the number of components and phonological control of the stimuli).

## Conclusions

In summary, speech accuracy and sentence generativity can be achieved in children with CI from interventions that incorporate EBI and matrix training. Participants with CI and who were readers improved their speech accuracy when tacting pictures of scenes after EBI involving dictated sentences, pictures of scenes, and sentences construction. The results confirmed equivalence relations as a route to integrate reading (CD) and tacting (BD) (Mackay & Sidman, 1984; Sidman, 1986), replicating previous findings (Anastácio-Pessan et al., 2015; Lucchesi et al., 2015) and expanding to sentences. Teaching verbal relations to diagonal sentences from a matrix with subject-verb-object combinations helped children with CIs to tacting novel pictures of scenes, using matrix untrained sentences; this result corroborates previous studies with normal hearing and CIs populations (Frampton et al., 2016; (Goldstein, 1983a, 1983b; Goldstein et al., 1987; Golfeto & de Souza, 2015; Yamamoto & Miya, 1999). The findings in the present study can be incorporated into research and the rehabilitation of children with CIs and may serve as a basis for making decisions in clinical situations and in sentence curriculum proposal.

Finally, some issues of this study need further investigation. Phonological balance must be adjusted to control some difficulties (as consonant clusters) and to measure the impact on auditory recognition and speech production in children with CIs. Repetitions in training that involved matching pictures to dictated sentences training suggest that errorless learning procedures may be assessed to determine whether auditory-visual conditional learning with sentences is faster under such conditions, if compared to the trial and error procedure adopted in this study. Future investigations may refine construction tasks to promote adequate stimulus control topography (Matos et al., 2006). Given the potential of matrix training for children with CIs, we recommend research to manipulate matrix arrangements and to maximize recombinative generalization. Lastly, future studies should seek to replicate these findings with more children with CIs and with communication disorders, in other laboratories and clinical contexts.

## Abbreviations

CI: Cochlear implant
CPA/HRAC: Audiological Research Center
CRMTS: Constructed-response matching-to-sample
EBI: Equivalence-based instruction
ITI: Intertrial interval
MTS: Matching-to-sample
PPVT-4: Peabody Picture Vocabulary Test-IV
S-V-O: [subject]-[verb]-[object] structure

## Footnotes

1 This method, item identification, is adopted from studies in the area of speech-language Pathology that seek to measure participants’ speech intelligibility. This method involves the transcription of speech stimuli by the listener and assigning a score or percentage of correctly or incorrectly decoded items (Barreto & Ortiz, 2010).

2 Normal-hearing peers show a typical sequence of these expressive skills. They first acquire speech accuracy in tacts through auditory-visual learning with the verbal community. They then begin to read, which allows accurate speech that is based on textual stimuli.

3 Form A of the Peabody Test (Dunn & Dunn, 2007) was translated and adapted to Portuguese and used as a measure of children’s receptive vocabulary. This translated version was based on the Peabody Picture Vocabulary Test-III standardization that was conducted by Capovilla and Capovilla (1997).

4 Hearing categories ranged from 1 to 6 and were defined by clinical evaluation, Minimum Hearing Capacity Assessment Test, Infant Toddler: Meaningful Auditory Integration Scale (IT-MAIS), Procedure for the Assessment of Deeply Hearing Impaired Children, and List of day-to-day Portuguese sentences and Word list for the perception of speech sounds. Language categories ranged from 1 to 5 and were defined mainly by the Meaningful Use of Speech Scales (MUSS).
